# Draft Genome Sequence of *Grammothele lineata* SDL-CO-2015-1, a Jute Endophyte with a Potential for Paclitaxel Biosynthesis

**DOI:** 10.1128/genomeA.00825-17

**Published:** 2017-08-17

**Authors:** Avizit Das, Oly Ahmed, A. K. M. Abdul Baten, Samira Bushra, M. Tariqul Islam, Ahlan Sabah Ferdous, Mohammad Riazul Islam, Haseena Khan

**Affiliations:** a Department of Biochemistry and Molecular Biology, University of Dhaka, Dhaka, Bangladesh; b Southern Cross Plant Science, Southern Cross University, Lismore, New South Wales, Australia

## Abstract

*Grammothele lineata* strain SDL-CO-2015-1, a basidiomycete fungus, was identified as an endophyte from a jute species, *Corchorus olitorius* var. 2015, and found to produce paclitaxel, a diterpenic polyoxygenated pseudoalkaloid with antitumor activity. Here, we report the draft genome sequence (42.8 Mb with 9,395 genes) of this strain.

## GENOME ANNOUNCEMENT

*Grammothele lineata* strain SDL-CO-2015-1 is a basidiomycote belonging to the family *Polyporaceae*; it has been isolated as an endophyte of *Corchorus olitorius* var. 2015. Current studies in our lab have revealed that this strain has the potential to produce a diterpenic polyoxygenated pseudoalkaloid-paclitaxel (taxol), an antitumor drug, in culture conditions (1); however, the molecular mechanisms and pathways of taxol production in fungus have not been fully elucidated (2). In order to attain the genomic support for the existence of an independent paclitaxel biosynthesis pathway, we report here the draft genome sequence of *G. lineata* SDL-CO-2015-1.

The strain was cultured in potato dextrose agar (PDA) medium containing 50 ng/mL of ampicillin. Total genomic DNA was isolated using a modified CTAB-SDS DNA extraction protocol developed in our laboratory. Genomic DNA was sequenced using Illumina HiSeq and PacBio RSII with one SMRT cell (1st BASE, Malaysia). The quality of the reads was checked using FastQC (http://www.bioinformatics.babraham.ac.uk/projects/fastqc), and the adapter trimming was done with Cutadapt (3). Both Illumina reads and PacBio reads were utilized to maximize the contiguity of the resulting genome assembly sequences. The genome was assembled by the SPAdes assembler version 3.9.1 (4), which involved the following steps: multi-*k*-mer-based assembly; long-read error correction; and assembly and scaffolding of the contigs. Pilon version 1.16 (5) was used to polish the assembled data in order to remove small mismatch or insertion/deletion errors that are caused by the inherent biases of the sequencing platforms. The assembled genome has a total size of 42,856,078 bp, with 1,276 scaffolds and an *N*_50_ value of 169,521 bp. The longest scaffold has a length of 810,186 bp.

We used proteins and expressed sequence tags from three closely related species as references for evidence-based gene prediction. MAKER version 2-32 (6) identified 9,395 high-confidence gene models with an average gene length of 2,271.457 bp. BUSCO version 2 (7) was used to assess the completeness of the genome, which reported the genome to be 93.10% complete. InterProScan version 5.17-56.0 software (8) was used to search and assign gene ontology (GO) terms to these gene models; out of the 9,395 genes, 6,519 were found to have at least 1 GO term assigned to them (in total, 13,534 GO terms were assigned to 6,519 genes). The GC content of the genome was determined to be 56.8%. RepeatMasker (http://www.repeatmasker.org), with the latest version of the repbase database, was used to identify the known repetitive elements; 3.61% of the genome was found to be repetitive, and 2.8% of those repeats were unclassified, as they are newly identified repeats (*de novo*) specific to this species.

As the expected biosynthetic pathway for paclitaxel in fungi is expected to be different from that of plants (9), our reported genomic data of *G. lineata* SDL-CO-2015-1 will be of colossal importance in understanding taxol production and metabolic manipulation in this strain in particular and fungi in general.

### Accession number(s).

This whole-genome shotgun project has been deposited at DDBJ/ENA/GenBank under the accession number NDFF00000000. The version described in this paper is the second version, NDFF02000000.
